# Accuracy, Hemorrhagic Complications and CT Radiation Dose of Emergency External Ventricular Drain (EVD) Placement in Pediatric Patients: A 15-Year Retrospective Analysis

**DOI:** 10.3390/diagnostics13172805

**Published:** 2023-08-30

**Authors:** Robert Stahl, Melvin D’Anastasi, Robert Forbrig, Thomas Liebig, Sophie Katzendobler, Jonathan Weller, Christoph G. Trumm

**Affiliations:** 1 Institute for Diagnostic and Interventional Neuroradiology, LMU University Hospital, LMU Munich, Marchioninistr. 15, 81377 Munich, Germany; robert.forbrig@med.uni-muenchen.de (R.F.); thomas.liebig@med.uni-muenchen.de (T.L.); christoph.trumm@med.uni-muenchen.de (C.G.T.); 2 Medical Imaging Department, Mater Dei Hospital, University of Malta, MSD 2090 Msida, Malta; melvin.a.danastasi@gov.mt; 3 Department of Neurosurgery, LMU University Hospital, LMU Munich, Marchioninistr. 15, 81377 Munich, Germany; sophie.katzendobler@med.uni-muenchen.de (S.K.); jonathan.weller@med.uni-muenchen.de (J.W.)

**Keywords:** external ventricular drain, pediatrics, accuracy, complications, radiation dose

## Abstract

Purpose: To assess accuracy, the frequency of hemorrhagic complications and computed tomography (CT) radiation dose parameters in pediatric patients undergoing landmark-guided external ventricular drain (EVD) placement in an emergency setting. Methods: Retrospective analysis comprised 36 EVD placements with subsequent CT control scans in 29 patients (aged 0 to 17 years) in our university hospital from 2008 to 2022. The position of the EVD as well as the presence and extension of bleeding were classified according to previously established grading schemes. Dose length product (DLP), volume-weighted CT dose index (CTDI_vol_) and scan length were extracted from the radiation dose reports and compared to the diagnostic reference values (DRLs) issued by the German Federal Office for Radiation Protection. Results: After the initial EVD placement, optimal positioning of the catheter tip into the ipsilateral frontal horn or third ventricle (Grade I), or a functional positioning in the contralateral lateral ventricle or the non-eloquent cortex (Grade II), was achieved in 28 and 8 cases, respectively. In 32 of 36 procedures, no evidence of hemorrhage was present in the control CT scan. Grade 1 (<1 mL) and Grade 2 (≥1 to 15 mL) bleedings were detected after 3 and 1 placement(s), respectively. For control scans after EVD placements, CTDI_vol_ (median [25%; 75% quartile]) was 39.92 [30.80; 45.55] mGy, DLP yielded 475.50 [375.00; 624.75] mGy*cm and the scan length result was 136 [120; 166] mm. Exceedances of the DRL values were observed in 14.5% for CTDI_vol_, 12.7% for DLP and 65.6% for the scan length. None of these values was in the range requiring a report to the national authorities. Conclusion: Landmark-based emergency EVD placement in pediatric patients yielded an optimal position in most cases already after the initial insertion. Complications in terms of secondary hemorrhages are rare. CT dose levels associated with the intervention are below the reportable threshold of the national DRLs in Germany.

## 1. Introduction

External ventricular drain (EVD) placement is defined as the percutaneous insertion of a (silicone polymer) catheter into the ventricular system. It is considered one of the most common minimally invasive neurosurgical procedures, even being one of the first procedures to be learned and independently carried out by neurosurgical residents in the operating or emergency room, or as a bedside intervention [1,2,3,4]. Emergency EVD placement is frequently performed for the treatment and monitoring of acute hydrocephalus and elevated intracranial pressure (ICP) due to intracranial bleeding (particularly intraparenchymal, intraventricular or subarachnoid hemorrhage), edema after ischemic stroke, tumors or severe traumatic brain injury (TBI) [5,6,7]. The most commonly used right frontal approach through a twist-drill burr hole at Kocher’s point is traditionally performed with a landmark-based technique and has several advantages such as easy patient positioning, a passage through the non-dominant hemisphere, a wide distance from the eloquent cortex, and—depending on patients’ anatomy—a relatively large target region of the frontal horn of the ipsilateral lateral ventricle [2]. The most common immediate and delayed major complications of EVD placement include hemorrhage and infection, respectively, often requiring repositioning or replacement [8,9]. Although the precision of placement is critical with respect to avoiding bleeding complications and tissue damage [10], the above-mentioned landmark-based or even a freehand technique [11] is still widely preferred in spite of the availability of alternative technical solutions for imaging guidance [12] or stereotactic navigation [13], particularly in the acute setting.

In pediatric patients, EVD implantation is performed using the aforementioned freehand method in the same way as in adults. However, the available literature on its indications and complication rates is still limited, not to mention evidence-based recommendations within guidelines, particularly the use of computed tomography (CT) for monitoring ICP and verification of correct EVD positioning [13,14,15,16]. In 2022, a revision of the diagnostic dose reference levels (DRL) issued by the German Federal Office for Radiation Protection was published [17]. Compared to the previous version from 2016, the age-dependent DRLs have been reduced again, in some cases significantly, which highlights the importance of radiation protection in pediatric imaging. Furthermore, for CT imaging, DRLs were specified for the parameters CTDI_vol_ and DLP in the old version. In contrast, the new version omits the DRLs for DLP. Instead, a scan length typical for the particular examination is specified.

Until now, the retrospective analyses by Ngo et al. and Miller et al. are the largest studies to report the clinical indications and complication rates of EVD placement in 66 and 63 pediatric individuals, respectively [15,16]. However, both reports did not focus on CT radiation doses. In comparison, a retrospective case–control study on 34 adults by Nowacki et al. [18] investigated two techniques of bolt-kit EVD placement, either a landmark-based (with control CT after EVD insertion) or CT-guided (with dose-reduced planning CT scan after bolt fixation and control CT scan after EVD insertion) approach, providing comparative data on cumulative CT radiation dose associated with both techniques.

In the above-mentioned context, our retrospective study aimed to analyse (i) the clinical indications; (ii) the accuracy of the landmark-based standard technique; (iii) the frequency of bleeding complications; as well as (iv) the CT radiation dose parameters against the background of the revised DRL levels in Germany in a cohort of pediatric patients having undergone emergency EVD placement by neurosurgeons in our university hospital.

## 2. Materials and Methods

### 2.1. Patients

**Figure 1:**
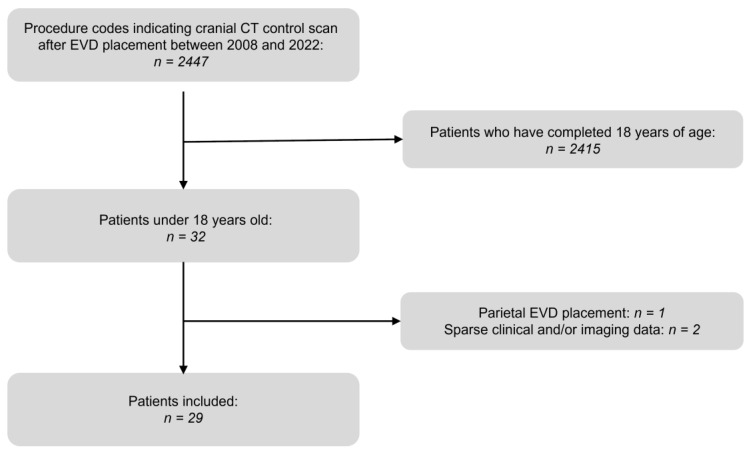
Patient selection chart.

The local ethics committee approved this retrospective study (number 23-0419). The Health Insurance Portability and Accountability Act standards for the privacy of personal health information as well as the ethical principles of the Declaration of Helsinki were followed. Our study comprised all pediatric patients who had undergone landmark-based EVD placement at our institution between January 2008 and December 2022. To define the study population, a Radiology Information System (RIS) database analysis was carried out yielding a total of 2447 EVD placement procedures with at least one cranial CT scan performed as part of the immediate post-procedural workup available in the local PACS. A sub-analysis yielded a total of 29 individuals having been admitted to our neurosurgery department during the study period in an emergency setting for the purpose of placing at least one EVD, summing up to a total of 36 procedures. Details of the selection process are depicted in Figure 1.

### 2.2. Neurosurgical Technique

EVD placements were generally performed in the CT scanner room or the operating theatre by neurosurgeons with board certification or equivalent experience, applying an established neurosurgical technique as previously described [18,19]. This usually involved the primary localization of Kocher’s point on the right side [19]. Depending on patient anatomy, the point was located 11.0–12.5 cm posterior to the nasion and 2.0–3.0 cm lateral to the midline. After determining Kocher’s point, skin disinfection with povidone–iodine and surgical draping ensued, followed by a skin incision of approximately 5 mm. Then, a burr-hole trephination was performed with a manually operated twist drill. The trajectory for EVD insertion was determined by the ipsilateral medial canthus in the coronal plane and the external auditory meatus in the sagittal plane. The EVD catheter with stylet was introduced to a depth of 3 to 4 cm. Then, the stylet was removed and the catheter was inserted into a depth of no more than 5 to 7 cm in patients with adult-sized heads, or to an appropriate depth according to the size of the child’s head, as determined individually in a 3-dimensional reconstruction prior to EVD placement [15,18]. If no CSF could be aspirated, a CT scan was acquired in order to document the catheter position. In case of incorrect placement, the catheter was removed and a new EVD catheter was inserted using an adjusted angulation. In case of a too-short distance of the catheter tip from the ventricular frontal horn or a too-deep catheter insertion into the ventricular system, the catheter was further inserted or withdrawn, respectively. In all cases, a final postprocedural CT control scan was acquired to verify the correct catheter position and to rule out immediate complications. EVD placement in the left hemisphere was only performed in cases of interfering pathologies on the right hemisphere, such as intracerebral hemorrhage, infarction or tumors in close proximity to the planned EVD trajectory, subdural hematoma, skull fractures, history of right-sided decompressive hemicraniectomy, or a unilateral slit ventricle caused by elevated intracranial pressure in the right hemisphere. Bilateral EVD placement was evaluated in case of pathologies in the third ventricular and/or occlusion of the foramina of Monro.

### 2.3. Imaging

Depending on the individual clinical case, the planning scan for the EVD placement was done either by acquiring a head CT scan in the same session in which the EVD placement took place or by using a preceding head CT/MR or ultrasound examination on another device. After EVD insertion, at least one CT scan was conducted as described above. In all patients a dedicated low-dose head CT protocol was applied, utilizing dose reduction regimens such as iterative reconstruction (e.g., SAFIRE and ADMIRE; Siemens Healthineers, Erlangen, Germany) and improved detector technology (e.g., Stellar Infinity detector with improved signal-to-noise ratio (SNR) and optimization of dose efficiency; Siemens Healthineers) whenever available. Scanners that had any of the above-described techniques were considered a “new” generation in our analysis.

### 2.4. Study Analysis

Radiology reports and clinical charts were reviewed with regard to patient characteristics and to identify the underlying disease requiring an EVD placement. The cranial head scans performed immediately before and after EVD placement archived in the local PACS were investigated to analyze the following items.

The EVD position after each placement trial was graded according to Kakarla et al. [1] (I = optimal placement of catheter tip into the ipsilateral frontal horn or third ventricle; II = functional placement in the contralateral lateral ventricle or non-eloquent cortex; III = suboptimal placement in the eloquent cortex or non-target CSF space). 

The presence and extension of intracranial hemorrhage following final EVD placement was classified as suggested by Wiesmann and Mayer [20] (Grade 0 = no bleeding; Grade 1: <1 mL; Grade 2: ≥1 to 15 mL; Grade 3: ≥15 mL). 

Assessment of the ventricular width as well as the presence and amount of midline shift were conducted as previously performed by Nowacki et al. [18].

CT dosimetric data in terms of CTDI_vol_, DLP, and scan length were extracted from the DICOM dose report of the PACS. In addition, these parameters were documented separately for the planning scans (CT scans prior to EVD insertion) and for the control scans (CT scans during or after the final placement of EVD). For each dose parameter, the number of values above the DRL threshold was determined. The effective dose was calculated by multiplying DLP values by a conversion factor according to Deak et al. [21]. Furthermore, an “excess factor” was calculated by dividing the current value of each parameter by the value of the respective DRL. Excess factors greater than 1 thus indicate the amount the DRL was exceeded in each case.

The control scan is the most important CT series during an emergency EVD placement since it is always performed at least one time, regardless of whether a previous CT planning scan is conducted or not. However, depending on the clinical requirements it can be acquired in varying numbers per insertion. In order to determine a representative dose sample from the control scans, we determined the mean DLP of the control scans within each session. From these resulting mean DLPs, the effective dose was calculated as described above. To compare the mean DLP of the control scans and its resulting effective dose between younger and elder children, we divided our collective into age groups of 0–11 years (child) and 12–17 years (adolescent).

### 2.5. Statistical Analysis

Count data as well as categorical data are reported as counts and percent. Continuous data were initially assessed for normality using the Shapiro–Wilk test and by visual inspection of their histograms. Normally distributed variables are provided as mean ± standard deviation. Variables that do not follow normal distribution are shown as median [25%; 75% quartiles]. For the latter two, the range was also given.

Mann–Whitney tests were used to determine significant differences in continuous radiation dose data and its derived excess factor between planning and control scans and between the scanner generations. Group differences in the amount of values exceeding the respective DRL were assessed with four-field tables and Fisher exact tests. When appropriate, a Bonferroni correction was applied to account for multiple comparisons. To assess differences in DLP and effective dose between the two age groups Mann–Whitney tests were applied.

Analysis was performed using R (R Core Team (2022). R: A language and environment for statistical computing. R Foundation for Statistical Computing, Vienna, Austria. URL https://www.R-project.org/, version 4.2.2, accessed on 15 April 2023). A level of significance of alpha = 0.05 was used throughout the study.

## 3. Results

### 3.1. Patient Collective

Baseline characteristics are shown in Table 1. In total, 29 individuals (12 female, 17 male patients; 0 to 17 years; mean age 11.4 ± 5.1 years) having undergone emergency EVD placement in our university hospital from 2008 to 2022 were identified according to the inclusion criteria and included in the study analysis. A total of 23 patients had one intervention and in 5 patients, 2 interventions were performed. In one patient, three EVD placements were conducted.

The majority of patients were admitted due to an intracranial tumor (n = 8), followed by TBI and non-traumatic intracranial hemorrhage (n = 7 each). Six patients received an EVD for acute dysfunction of a preexisting ventriculo-peritoneal or ventriculo-atrial shunt. These shunts were inserted for hydrocephalus in a posthemorrhagic (n = 4), postinfectious (n = 1) or congenital (n = 1, Arnold Chiari malformation type 2) condition. One EVD placement was conducted because of a malignant infarction of the vertebrobasilar system.

### 3.2. Perinterventional Analysis

#### 3.2.1. Intervention Situs

A right, left and bilateral EVD access was chosen in 26, 7 and 3 procedures, respectively (Table 2). Ventricular width was 13.4 ± 6.5 (2 to 26) mm. In 8/36 sessions midline shift (MLS) was present. The mean amount of the MLS was 4.9 ± 3.6 mm.

#### 3.2.2. Imaging

In 21 (58.3%) sessions a previous cross-sectional imaging study was available and a dedicated planning CT scan during the same session was omitted before EVD insertion. Conversely, in 15 (41.7%) sessions a planning CT scan was acquired.

The examinations were performed on the following CT scanners: SOMATOM Definition AS+ (n = 16 (44.4%); 128 detector rows; Siemens Healthineers; new generation); BrightSpeedS (n = 7 (19.4%); 16 rows; General Electric; old generation); SOMATOM Force (n = 7 (19.4%); 2 × 192 detector rows; Siemens Healthineers; new generation); SOMATOM Definition Edge (n = 3 (8.3%); 128 detector rows; Siemens Healthineers; new generation); SOMATOM Definition Flash (n = 1 (2.8%); 2 × 128 detector rows; Siemens Healthineers; old generation); SOMATOM Sensation 64 (n = 1 (2.8%); 64 detector rows; Siemens Healthineers; old generation); and SOMATOM Drive (n = 1 (2.8%); 128 detector rows; Siemens Healthineers; new generation).

The procedure time between the first and last CT scan was median [25%; 75% quartiles] 22 [15; 52] (5 to 94) min. Number of control CT scans after EVD placement was 1 [1; 2] (1–4). This comprises 21/13/0/2 sessions with 1/2/3/4 control CT scans, respectively.

#### 3.2.3. Accuracy

In a total of 36 procedures, a final Kakarla I and II EVD position was achieved in 35 (97.2%) and 1 (2.8%) sessions, respectively (Table 3).

**Figure 2:**
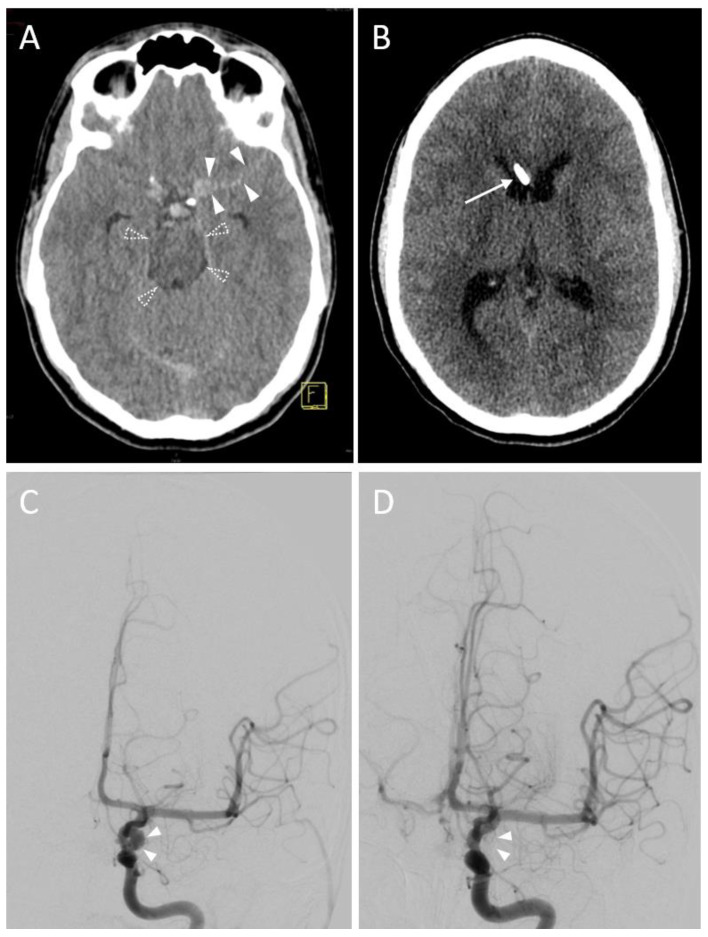
A 16-year-old male with a subarachnoid hemorrhage from an aneurysm of the left internal carotid artery. (**A**) Planning a CT scan revealed a subarachnoid hemorrhage (Fisher Grade IV). Blood collections are shown in the perimesencephalic cistern (transparent arrowheads) and the lateral fissure (opaque arrowheads). Due to beginning hydrocephalus, indication for EVD placement was given. Yellow graphic: orientation cube (f: frontal). (**B**) Control CT scan. Successful EVD placement in the right frontal horn with tip next to the septum pellucidum (Kakarla I). (**C**) Digital Subtraction Angiography (DSA) before endovascular treatment. Source of bleeding is a broad-based saccular aneurysm at the outlet of the left posterior communicating artery with a maximum diameter of 6.5 × 5.0 mm (arrowheads). (**D**) DSA control scan after coil placement depicts complete elimination of the aneurysm sac.

In the majority of sessions (n = 28, 77.8%), a Kakarla I EVD position was documented after the first control CT scan (Figure 2).

**Figure 3:**
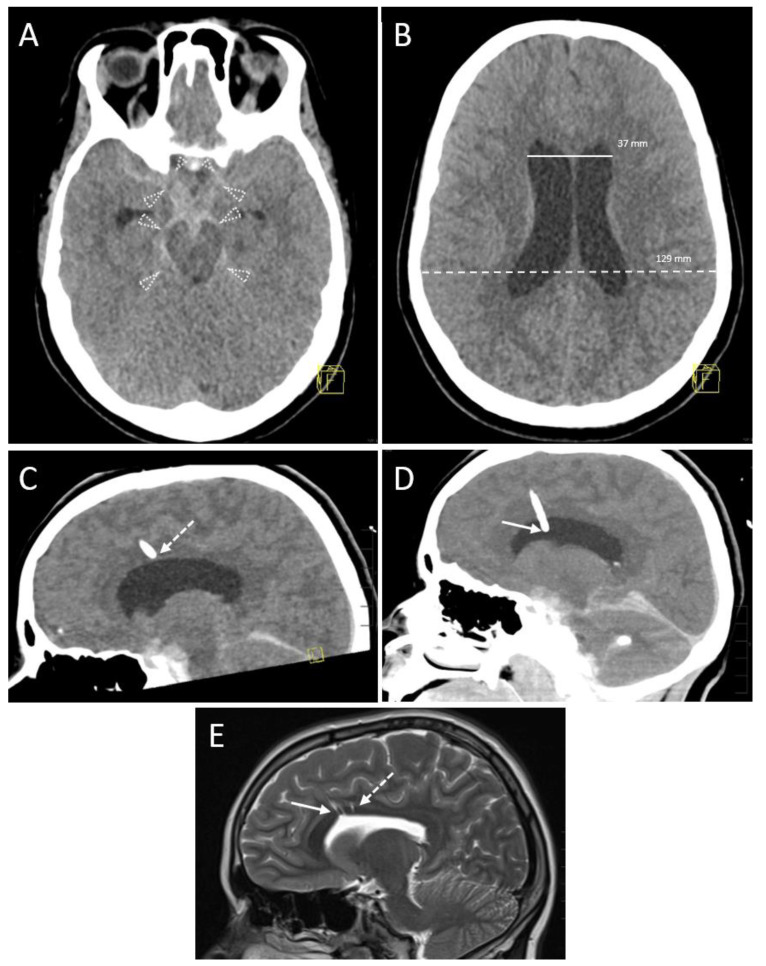
A 14-year-old female with a subarachnoid hemorrhage (Fisher Grade IV) after a motor vehicle accident. (**A**) Planning a CT scan revealed blood collections in the pentagonal and perimesencephalic cisterns (arrowheads). Yellow graphic: orientation cube (f: frontal). (**B**) The Evans index of 0.29 (ratio of the maximum width of the frontal horns of the lateral ventricles (37 mm, solid line) and the maximal internal diameter of the skull (129 mm, dashed line)) was in borderline range, suspicious for slightly increased ventricular width. Consequently, the indication for EVD placement was given. Yellow graphic: orientation cube (f: frontal). (**C**) After EVD placement aspiration of cerebrospinal fluid was not possible. Sagittal reconstruction of the first control scan showed a malposition of the drainage tip in the anterior body of the corpus callosum (dashed arrow). Yellow graphic: orientation cube. (**D**) Sagittal Maximum Intensity Projection (MIP; 6 mm slice thickness) of the control scan after repositioning of the EVD. The drainage tip is now correctly placed in the anterior horn of the lateral ventricle (solid arrow). (**E**) A control MRI scan 2 years later shows the puncture channels of the EVD malposition (dashed arrow) as well as their correction placement (solid arrow) as parenchymal defects.

However, in 11 (30.6%) sessions the neurosurgeon decided to proceed with applying either further EVD insertion (n = 5, 13.9%) or withdrawal (n = 1, 2.8%) or modification (n = 5, 13.9%) of the EVD position in case of inadvertent touching or penetration of critical structures or impossible aspiration (head (n = 1) or body (n = 1) of caudate nucleus, internal capsule (n = 1), knee of corpus callosum (n = 1, Figure 3) and septum pellucidum towards contralateral ventricle (n = 1).

Only in two (5.6%) sessions four control CT scans were required to ensure a functioning final EVD position, one of them remaining a Kakarla II EVD position within the frontal horn of the contralateral ventricle.

#### 3.2.4. Hemorrhage

**Figure 4:**
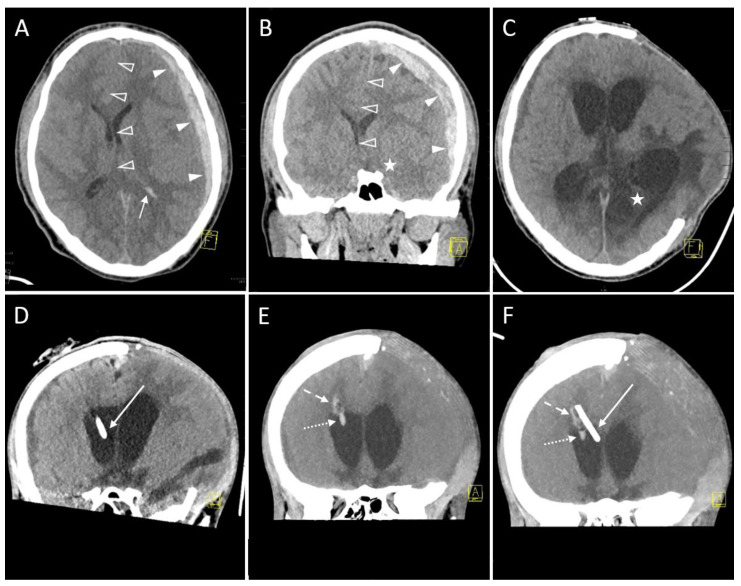
A 16-year-old boy after a motorbike accident. Axial (**A**) and coronal reformation (**B**) of the CT scan acquired immediately after the trauma revealed traumatic subdural hematoma (solid arrowheads), midline shift to the right (transparent arrowheads), uncal herniation (asterisk) and traumatic subarachnoid bleeding because of blood collection in the posterior horn of the left ventricle (arrow). A left-sided decompressive trepanation was conducted. Yellow graphic: orientation cube (a: anterior, f: frontal). (**C**) After one month, a posttraumatic hydrocephalus developed with increasing width of the ventricles. Thus, the indication for an EVD placement was made. Note the infarction of the posterior cerebral artery territory which had occurred due to the herniation and which is now clearly demarcated (asterisk). The left hemispheric brain exceeds the trepanation margins. Onset of general brain atrophy could also be seen. Yellow graphic: orientation cube (f: frontal). (**D**) Coronal Maximum Intensity Projection (MIP; 4 mm slice thickness) of the control scan after EVD placement shows the tip in the frontal horn of the right ventricle (Kakarla I). Yellow graphic: orientation cube (a: anterior). (**E**) However, after 3 days the EVD has been removed due to clotting (dotted arrow) and intraparenchymal hemorrhage (dashed arrow; volume ≥ 1 to 15 mL, Grade 2 according to Wiesmann and Mayer). Yellow graphic: orientation cube (a: anterior). (**F**) Coronal Maximum Intensity Projection (MIP; 20 mm slice thickness) of the control scan after placement of the new EVD. Its tip (solid arrow) is located near the septum pellucidum (Kakarla I). Clotting (dotted arrow) and intraparenchymal hemorrhage (dashed arrow) caused by the former EVD are still visible. Yellow graphic: orientation cube (a: anterior).

According to the classification by Wiesmann and Mayer [20], after 32 (88.9%) interventions, there was no evidence of hemorrhage in the immediate control CT scan and remaining follow-up CT scans acquired during the first 7 days after EVD placement (Table 3). Conversely, Grade 1 and 2 (Figure 4) hemorrhages were seen after 3 (8.3%) and 1 (2.8%) session(s), respectively. Grade 3 bleedings did not occur (0.0%).

#### 3.2.5. CT Radiation Dose

The total cumulative dose length product of all 36 EVD placement sessions with (n = 15) or without (n = 21) a dedicated planning CT scan was 1064 [671, 1399] mGy*cm (117 to 10,321). The cumulative DLP of sessions with planning CT scans (n = 15) was 1307 [1118, 1612] mGy*cm (446 to 10,321), while the cumulative DLP of sessions with control CT scans only (n = 21) was 820 [426, 1151] mGy*cm (117 to 5575). Correspondingly, the total cumulative effective dose of all 36 EVD placement sessions (with or without a dedicated planning CT scan) was 2.55 [1.66, 3.40] (0.41–19.60) mSv. Cumulative effective dose of sessions with planning CT scans was 3.16 [2.45, 3.45] (1.56–19.60) mSv, while cumulative effective dose of sessions with control CT scans only was 1.89 [1.20, 3.11] (0.41–10.60) mSv.

DLP values and its excess factor were significantly (*p* < 0.05) higher in the planning scans compared to the control scans (DLP values (median [25%, 75% quartile]): planning scan: 600.22 [492.06; 747.85] mGy*cm vs. control scan: 475.50 [375.00; 624.75] mGy*cm; DLP excess factor: planning scan: 0.77 [0.70; 0.99] mGy*cm vs. control scan: 0.69 [0.49; 0.79] mGy*cm; Supplementary Table S1). CTDI_vol_, scan length and their derived indicators were also slightly elevated in the planning scans compared to the control scans. However, this difference was not statistically significant (*p* > 0.05).

**Figure 5:**
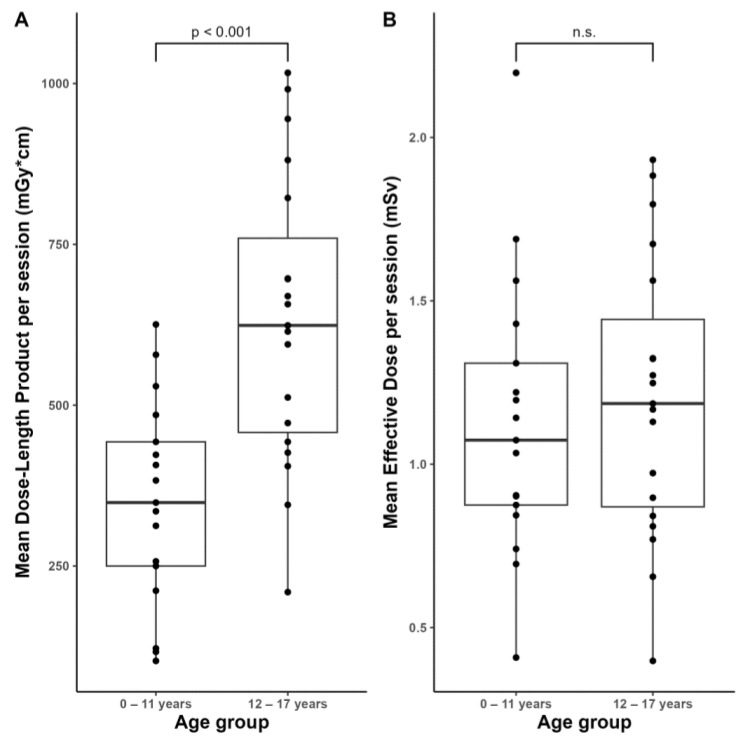
Distribution of the averaged dosimetry data of the control scans per session across the two age groups.(**A**) Mean Dose-Length Product. (**B**) Mean Effective Dose. n.s.: not significant (*p* > 0.05).

The median values of the control scans averaged per intervention were 458 [343, 633] (102–1017) mGy*cm for DLP and 1.15 [0.87, 1.35] (0.40–2.20) mSv for the effective dose. A comparison of the age groups yielded significantly (*p* < 0.001) higher DLP values of the elderly compared to the younger ones (Figure 5A: age group 0–11 years: 349 [250, 443] (102–626) mGy*cm vs. age group 12–17 years: 624 [458, 760] (209–1017) mGy*cm), but only tendentially (*p* > 0.05) higher effective dose values (Figure 5B: age group 0–11 years: 1.07 [0.89, 1.31] (0.43–2.20) mSv vs. age group 12–17 years: 1.19 [0.89, 1.44] (0.40–1.93) mSv).

Comparing old and new scanner generations, the scan length tended (*p* > 0.05) to be longer for new devices than for older devices in both planning and control scans. In planning scans, CTDI_vol_ tended (*p* > 0.05) to be higher in old devices compared to new scanners. In contrast, CTDI_vol_ and its excess factor were slightly (*p* > 0.05) higher for new devices in control scans, while the DRL was exceeded marginally (*p* > 0.05) less often in new scanners.

DLP values tended (*p* > 0.05) to be higher for new scanners in both types of series than for old devices. This also applied to the DLP excess factor, whereby this group difference was statistically significant (*p* < 0.05) in control scans (old scanners: 0.48 [0.36; 0.52] mGy*cm vs. new scanners: 0.71 [0.54; 0.79] mGy*cm). However, the DRL for DLP was exceeded slightly (*p* > 0.05) more often in old devices for both types of series.

#### 3.2.6. Follow-up

The time to removal (TTR) of the EVD overall studied patients was 6 [3; 14.2] (1–43) days. Seven patients died during the current hospital stay (TTR: 4 [2.5; 6] (1–9) days). In 14 patients a ventriculo-atrial or a ventriculo-peritoneal shunt was inserted (TTR: 9 [5; 25] (1–43 days). TTR in patients who did not receive a shunt (and excluding those who died during admission) was 10 [3.5, 14.5] (2–25) days. None of the EVDs analyzed in this study showed evidence of superinfection.

## 4. Discussion

Our study focused on the accuracy, hemorrhage rate and analysis of CT radiation dose in an unselected pediatric patient population undergoing EVD placement utilizing a widely known landmark-based neurosurgical technique.

When analyzing the accuracy of EVD insertion, the grading system by Kakarla et al. has been widely established [1]. In our study, a final Kakarla I (‘optimal placement of catheter tip into ipsilateral frontal horn or third ventricle’) EVD position was achieved in 35 of 36 procedures (97.2%), whereas in the remaining EVD placement sessions, a Kakarla II position (‘functional placement in contralateral lateral ventricle or non-eloquent cortex’) was documented in the final control CT scan. On the other hand, the misplacement rate at the first approach was 13.9% with five inadvertent primary EVD insertions into important anatomical structures (caudate nucleus, internal capsule, corpus callosum and septum pellucidum). A large number of authors investigated the accuracy of EVD placement with a slightly varying focus between the primary and secondary questions of the different studies [9,11,22,23]. In patients with acute hydrocephalus, both a real ‘freehand technique’ as well as a landmark-based technique (using a tape measure to identify the Kocher’s point for placement of the frontal burr hole) seem to be comparable with respect to the rates of correct positioning vs. malposition and intracerebral and/or intraventricular bleeding [11]. Percutaneous needle trephination (PNT) as well as conventional EVD insertion are also effective in the setting of acute hydrocephalus [20,24]. However, in a mixed patient population before EVD insertion, attention must be given to potential distortion of anatomy due to brain shift due to tumors, intracerebral hemorrhage (ICH), posttraumatic brain edema, large decompressive hemicraniectomy, etc. [23]. In this context, the retrospective study by Fisher et al. comprising 294 patients provided valuable insights into the added value of neuronavigation or image guidance [25]. In their study, a total of 183 catheters were placed freehand, 66 under neuronavigation guidance and 45 under ultrasound guidance. Given a comparable Evans index between the three patient subgroups, the authors found a tendency for lower rates of suboptimal placement when some guidance was utilized (20.8% freehand; 15.6% ultrasound; 10.6% neuronavigation). The authors concluded that, at least in selected cases, image-guided EVD placement is recommended to reduce misplacement and complication rates. Although the available literature underlines that in case of a lack of available residents, EVD insertion can be safely performed by midlevel practitioners, physician assistants and nurse practitioners [26,27]; a large armamentarium of auxiliary means is meanwhile clinically available that addresses abnormal or pathological ventricular anatomy and, therefore, have the potential to significantly reduce futile freehand/landmark-based attempts to introduce a functioning EVD and to reduce EVD misplacement rates [28,29,30,31,32,33,34,35,36,37]. Using neuronavigation can reduce radiation dose. However, neuronavigation systems are characterized by several drawbacks such as increased preparation time [38] for the EVD placement procedure mainly due to the registering of preinterventional scans, limited availability in the emergency room, the potential need for head fixation within Mayfield skull clamps depending on the locally available system which, in turn, cannot be inserted into the thin bones of children without risk. Headbands [31] and other frameless navigation systems [13,39] might be an alternative. Also, neuronavigation does not guarantee correct EVD positioning [13]. In contrast, completing the EVD placement procedure in the emergency room including a control CT scan avoids patient transportation inducing ICP peaks. Given the low number of pediatric patients requiring an EVD, time and cost efficiency must be weighed carefully against benefits for the patients. Although not clinically relevant in most cases, the brain tissue damage occurring along the trajectory after EVD placement becomes particularly evident when MRI is used during hospitalization, depicting not only very small hemorrhages but also intra- and extracellular edema along the catheter [10]. This makes optimization of the infrastructural prerequisites and sufficient staff training for EVD placement in the pediatric population even more mandatory.

Intracranial bleeding is one of the most common and significant potential complications of EVD placement, and Wiesmann and Mayer were forerunners to investigate this adverse event while introducing an easy-to-handle grading system for EVD-related hemorrhages [20]. Since then, numerous studies from monocentric retrospective case series to meta-analyses have analyzed hemorrhage rates after EVD insertion [8,19,40,41,42,43,44]. According to these, the incidence of hemorrhage (as calculated per inserted EVD) ranges between 0 [45] and 32.5% [43]. The meta-analysis by Binz et al. comprised 13 studies (each with more than 25 EVD insertions) conducted between 1980 and 2008, and reported hemorrhagic complications in 5.7% of 1790 EVD placements, with 0.61% being clinically significant (i.e., leading to a new neurological deficit, death or requiring surgical intervention) [40]. Comparing studies with vs. studies without routine post-procedure CT scans in a sub-analysis, hemorrhage rates were 10.06% vs. 1.53% (*p* < 0.001), whereas the rate of clinically significant ICH was not statistically different (0.91% vs. 0.33%, *p* = 0.113) between both study groups. This indicates a higher detection rate of asymptomatic ICH when a post-procedural CT is routinely acquired. As already underlined by Miller and Guillaume [15], in the pediatric population there are only a few studies that have investigated the frequency of EVD-related hemorrhage, with published rates up to 17.6% [16,46,47,48,49,50]. The hemorrhage rate observed in our patient series, calculated per EVD placement procedure (4/36 procedures; 11.1%), is in the range of the aforementioned studies. Of these four sessions with intraparenchymal hemorrhage being present after insertion, there were three Grade 1 (<1 mL) and one Grade 2 (≥1 to 15 mL) bleeding. In the latter case, the initially inserted EVD was exchanged three days later by a new EVD catheter after clotting (Figure 3). In terms of the above-mentioned definition of clinical relevance by Binz et al. [40], in our patient series, there was one of four EVD-related ICH with a consecutive neurosurgical intervention (=exchange of EVD), corresponding to a rate of 2.8% (1/36) of symptomatic EVD-related bleeding complications, while in the remaining cases, a functioning final EVD position without neurological sequelae was achieved. Both of the largest available studies evaluating the outcome of pediatric EVD by Ngo et al. [16] and Miller and Guillaume [15] reported hemorrhage rates of 4.2% and 10%, respectively, compared to an 11.1% rate (4 patients with 36 procedures) in our patient series. In addition to the established grading system used in previous studies [19,20], the detailedness varies markedly, with some authors simply describing the presence of hemorrhage as ‘any blood in the area of the branch canal’ [51] versus even calculating absolute ICH volume [15,43,52]. From our point of view, the grading system by Wiesmann and Mayer is particularly advantageous with respect to robustness and comparability against the results of other studies [20].

Another important safety aspect of the clinical EVD placement workflow is imaging for assessment of anatomy, planning of the trajectory and control of correct EVD placement. Although computed tomography is widely available and the imaging modality of choice for the assessment of bleeding complications, it implies the exposure of patients to ionizing radiation, which is particularly problematic in the pediatric patient population with nearly 1/3 of the whole hematopoietically active bone marrow in the skull of a newborn vs. 1/10 in a 15-year-old adolescent [53].

The cumulative DLP and effective dose as well as the procedure time observed in our study were in the range of the study by Nowacki et al. [18] who compared two different EVD placement methods (landmark-based vs. CT-guided insertion).

Comparing the different types of series used during EVD placement in our patients, we found that in the planning scans DLP was significantly higher and CTDI_vol_ and scan length tended to be higher than in the control scans. One explanation for this may be that in the planning CT scan a comparably larger scan range is usually selected to be able to assess the entire anatomical situation as in most cases it is an examination performed under emergency conditions. In contrast, the scanned volume in the control scan can be restricted to the area of the expected drainage path.

When our patient population was divided into two age groups, we found significantly higher DLP values in the elderly. This can be explained by the fact that the scan volume for smaller children is naturally lower than for older children. The effective dose, on the other hand, only tended to be higher in older children than in younger ones. Thus, we conclude that the biological impact of the use of ionizing radiation in EVD placement does not affect any age group in a particularly negative way.

Over the time course of our study, a dedicated low-dose CT protocol was increasingly performed, utilizing dose reduction regimens such as iterative reconstruction (SAFIRE and ADMIRE; Siemens Healthineers) and improved detector technology (i.e., Stellar Infinity detector with improved signal-to-noise ratio (SNR) and optimization of dose efficiency; Siemens Healthineers) in the modern CT scanners of the emergency unit. This was shown in our study by the fact that in the control scans the absolute values and excess factors for scan length, CTDI_vol_ and DLP were somewhat higher for new devices, but at the same time, the respective DRLs tended to be exceeded less often. We therefore conclude that—with the application of these technologies—compliance with the national DRLs is warranted more frequently. Overall, however, in the 15 years of our observation period, neither the use of equipment with old nor new CT technology showed that the respective applicable DRLs had been exceeded to such an extent that a report would have had to be made to the national authorities.

Our study is characterized by certain limitations: (i) The inclusion criteria of our study population (children with at least one control CT scan after EVD placement) resulted in patients who presented to the emergency department or who were already in a critical condition in the hospital because of acute hydrocephalus or increased intracranial pressure due to trauma, tumor or the acute exacerbation of vascular disease. The indication for CT was therefore made by an interdisciplinary team of neuroradiologists, neurosurgeons, and in some cases, trauma surgeons, because there was usually not enough time for elective follow-up scans with modalities that do not use ionizing radiation. Therefore, controlling the accuracy of the EVD position by CT is performed in our hospital only in exceptional cases. This approach reduced the number of patients ultimately included. (ii) The inclusion criteria yielded no patient in our study population who had an initial CSF infection as an indication for EVD placement. Both of these factors may mean that the results shown have limited validity. (iii) The CT radiation dose calculations were performed based on the DLP values provided in the DICOM dose report of each CT examination. (iv) The various CT scanners utilized for planning and control of EVD positioning represent the heterogeneous setting of a university hospital with partially substantial differences in the individual technical equipment.

## 5. Conclusions

Landmark-based EVD placement in pediatric patients yielded an optimal position in most cases already after the initial insertion. In addition, if the catheter is incorrectly positioned after the first attempt, it can usually be corrected during the same session.

Complications in the form of secondary hemorrhages are rare, usually self-limiting and have no further clinical sequelae.

The radiation dose values for the planning scan in the EVD placement are slightly higher than those for the control scans, since for the purpose of EVD insertion planning, the full anatomical situation usually has to be assessed. In contrast, in the control scan the scan length can be restricted to the expected drain course and length.

Even though older children have a larger DLP compared to younger ones due to head size, the biological radiation effect in terms of the effective dose does not increase significantly.

When radiation dose reduction technologies are applied, the national DRLs are exceeded tendentially less often. Regardless of the use of these technologies, national DRLs were not exceeded to the extent that reporting to the authorities would be required.

## Figures and Tables

**Table 1:** Baseline characteristics of the 29 patients.

Age (Years)	11.4 ± 5.1 (0–17) ^1^
Sex	
Female	12 (41.4) ^2^
Male	17 (58.6) ^2^
Indications for EVD placement	
*Tumor (WHO ^3^ grade)*	8 (27.6) ^2^
Pilocytic Astrocytoma (I)	1 (3.4) ^2^
Craniopharyngioma (I)	1 (3.4) ^2^
Ependymoma (III)	1 (3.4) ^2^
Glioblastoma (IV)	3 (10.3) ^2^
Medulloblastoma (IV)	1 (3.4) ^2^
Pineoblastoma (IV)	1 (3.4) ^2^
*Trauma*	7 (24.1) ^2^
Car accident	4 (13.9) ^2^
Motorbike accident	2 (6.8) ^2^
Pedestrian accident	1 (3.4) ^2^
*ICH ^4^ (non-traumatic)*	7 (24.1) ^2^
Arteriovenous Malformation	3 (10.3) ^2^
Aneurysm	1 (3.4) ^2^
Metastasis	1 (3.4) ^2^
Extracorporeal Membrane Oxygenation	1 (3.4) ^2^
Idiopathic thrombocytopenic purpura	1 (3.4) ^2^
*Other*	7 (24.1) ^2^
Acute dysfunction of VP-/VA ^5^ Shunt	6 (20.7) ^2^
Stroke	1 (3.4) ^2^

^1^: Mean value ± standard deviation (range), ^2^: numbers (percentage), ^3^: World Health Organization, ^4^: Intracerebral hemorrhage; ^5^: ventriculo-peritoneal/ventriculo-atrial.

**Table 2:** Information on intervention situs, side of drain placement and imaging in the 36 EVD placement procedures.

Ventricular Width (mm)	13.4 ± 6.5 (2–26) ^1^
Midline shift	
Present	8/36 (22.2) ^2^
Amount (mm)	4.9 ± 3.6 (2–12) ^1^
Drain insertion side	
Right	26/36 (72.2) ^2^
Left	7/36 (19.5) ^2^
Bilateral	3/36 (8.3) ^2^
Planning CT scan during same session	15 (41.7) ^2^
Additional CT scan same/previous day	5/2
Additional MRI scan same/previous day	1/4
No planning CT scan during same session	21 (58.3) ^2^
Additional CT scan same/previous day	14/4
Additional MRI scan same/previous day	1/0
Additional CT scan same or previous day	25 (69.4) ^2^
Additional MRI scan same or previous day	6 (16.7) ^2^
CT control scans per session	1 [1; 2] (1–4) ^3^
Sessions with a total of 1 control scan	21 (58.3) ^2^
Sessions with a total of 2 control scans	13 (36.1) ^2^
Sessions with a total of 3 control scans	0 (0.0) ^2^
Sessions with a total of 4 control scans	2 (5.6) ^2^
Procedure time (minutes)	22 [15; 52] (5–94) ^3^

^1^: Mean value ± standard deviation (range), ^2^: numbers (percentage), ^3^: Median [25%, 75% percentile] (range).

**Table 3:** Accuracy of EVD placement and hemorrhagic complications in 36 EVD placement procedures.

EVD position in 1st control CT scan	
Kakarla I	28 (77.8) *
Kakarla II	8 (22.2) *
Kakarla III	0 (0.0) *
EVD position in 2nd control CT scan	
Kakarla I	11 (30.6) *
Kakarla II	2 (5.6) *
Kakarla III	0 (0.0) *
EVD position in 3rd control CT scan	
Kakarla I	1 (2.8) *
Kakarla II	1 (2.8) *
Kakarla III	0 (0.0) *
EVD position in 4th control CT scan	
Kakarla I	1 (2.8) *
Kakarla II	1 (2.8) *
Kakarla III	0 (0.0) *
Final EVD position	
Kakarla I	35 (97.2) *
Kakarla II	1 (2.8) *
Hemorrhage on follow-up CT	
No evidence of hemorrhage	32 (88.9) *
Grade 1 (<1 mL)	3 (8.3) *
Grade 2 (≥1 to 15 mL)	1 (2.8) *
Grade 3 (>15 mL)	0 (0.0) *

*: numbers (percentage).

## Data Availability

The data presented in this study are available upon reasonable request from the corresponding author.

## Supplementary Materials

The following supporting information can be downloaded at: https://www.mdpi.com/article/10.3390/diagnostics13172805/s1. Table S1: Analysis of CT dosimetry data relevant for the DRLs from the 36 interventions.
