# Supplementary material for: Accuracy, Hemorrhagic Complications and CT Radiation Dose of Emergency External Ventricular Drain (EVD) Placement in Pediatric Patients: A 15-Year Retrospective Analysis
Source: Diagnostics (Basel). 2023 Aug 30;13(17):2805. doi: 10.3390/diagnostics13172805 (PMC10486425; doi:10.3390/diagnostics13172805)
Supplement: Supplementary file 1 [file diagnostics-13-02805-s001.zip › diagnostics-2528885-SI.pdf]

diagnostics

Supplementary Table S1: Analysis of CT dosimetry data relevant for the DRLs from the 36 interventions.

	Planning scans	p-value [1)]	Control scans	p-value [1)]	p-value [2)]
Scan length (mm)					
	155 [133; 173] (118-186)		136 [120; 166] (81-245)		0.121
- Old scanners	*133 [131; 156] (126-166)*	*0.038*	*126 [116; 128] (103-160)*	*0.117*	*0.047*
- New scanners	*171 [150; 175] (118-186)*		*146 [120; 174] (81-245)*		*0.171*
- Within DRL limits	2/19 (10.5%)		11/32 (34.4%)		0.231
- Old scanners	*2/8 (25.0%)*	*0.319*	*5/8 (62.5%)*	*0.212*	*0.223*
- New scanners	*0/11 (0.0%)*		*6/24 (25.0%)*		*0.180*
- Excess factor	1.25 [1.11; 1.35] (0.97-1.69)		1.09 [0.97; 1.36] (0.65-1.88)		0.198
- Old scanners	*1.13 [1.01; 1.34] (0.97-1.41)*	*0.247*	*0.98 [0.93; 1.07] (0.79-1.23)*	*0.061*	*0.112*
- New scanners	*1.32 [1.22; 1.35] (1.07-1.69)*		*1.18 [1.02; 1.48] (0.65-1.88)*		*0.384*
CTDI$_{vol}$ (mGy)					
	41.78 [32.81; 49.37] (4.80-59.62)		39.92 [30.80; 45.55] (7.27-60.26)		0.380
- Old scanners	*45.56 [32.27; 50.53] (4.80-59.62)*	*0.429*	*32.65 [24.88; 42.22] (9.72-59.62)*	*0.349*	*0.337*
- New scanners	*39.93 [34.02; 46.45] (21.77-56.97)*		*39.39 [31.17; 45.85] (7.27-60.26)*		*0.795*
- Within DRL limits	27/35 (77.1%)		47/55 (85.5%)		0.578
- Old scanners	*10/14 (71.4%)*	*0.685*	*9/11 (81.8%)*	*0.119*	*0.569*
- New scanners	*17/21 (81.0%)*		*38/44 (86.4%)*		*0.675*
- Excess factor	0.90 [0.71; 0.97] (0.19-1.32)		0.78 [0.71; 0.91] (0.28-1.28)		0.150
- Old scanners	*0.90 [0.86; 1.06] (0.19-1.32)*	*0.186*	*0.72 [0.49; 0.79] (0.28-1.08)*	*0.132*	*0.040*
- New scanners	*0.82 [0.70; 0.96] (0.62-1.13)*		*0.81 [0.72; 0.92] (0.29-1.28)*		*0.938*
DLP (mGy*cm)					
	600.22 [492.06; 747.85] (52.90-1184.70)		475.50 [375.00; 624.75] (93.00-1085.10)		**0.023**
- Old scanners	598.78 [482.88; 654.62] (52.90-897.00)	0.516	348.60 [296.14; 426.26] (116.64-945.00)	0.054	0.095
- New scanners	635.00 [539.00; 769.70] (233.60-1184.70)		529.50 [408.00; 627.70] (93.00-1085.10)		0.042
- Within DRL limits	27/35 (77.1%)		48/55 (87.3%)		0.251
- Old scanners	9/14 (64.3%)	0.220	9/11(81.8%)	0.617	0.406
- New scanners	18/21 (85.7%)		39/44 (88.6%)		0.706
- Excess factor	0.77 [0.70; 0.99] (0.18-1.39)		0.69 [0.49; 0.79] (0.25-1.36)		**0.003**
- Old scanners	0.77 [0.71; 1.05] (0.18-1.12)	0.606	0.48 [0.36; 0.52] (0.25-1.11)	**0.011**	0.035
- New scanners	0.80 [0.69; 0.96] (0.52-1.39)		0.71 [0.54; 0.79] (0.31-1.36)		0.029

[1)] p-values for comparison between old and new scanners within planning or control scans. [2)] p-values for comparison between planning and control scans. Values in bold indicate differences on the 5% level of significance. DRL: Diagnostic Reference Level. CTDI$_{vol}$: volume-weighted CT dose index. DLP: dose-length product. mGy: milligray.
